# Fibroblast fusion to the muscle fiber regulates myotendinous junction formation

**DOI:** 10.1038/s41467-021-24159-9

**Published:** 2021-06-22

**Authors:** Wesal Yaseen, Ortal Kraft-Sheleg, Shelly Zaffryar-Eilot, Shay Melamed, Chengyi Sun, Douglas P. Millay, Peleg Hasson

**Affiliations:** 1 Department of Genetics and Developmental Biology, The Rappaport Faculty of Medicine and Research Institute, Technion – Israel Institute of Technology, Haifa, Israel; 2 Division of Molecular Cardiovascular Biology, Cincinnati Children’s Hospital Medical Center, Cincinnati, OH USA; 3 Department of Pediatrics, University of Cincinnati College of Medicine, Cincinnati, OH USA

**Keywords:** Cell biology, Differentiation, Mesoderm, Transdifferentiation

## Abstract

Vertebrate muscles and tendons are derived from distinct embryonic origins yet they must interact in order to facilitate muscle contraction and body movements. How robust muscle tendon junctions (MTJs) form to be able to withstand contraction forces is still not understood. Using techniques at a single cell resolution we reexamine the classical view of distinct identities for the tissues composing the musculoskeletal system. We identify fibroblasts that have switched on a myogenic program and demonstrate these dual identity cells fuse into the developing muscle fibers along the MTJs facilitating the introduction of fibroblast-specific transcripts into the elongating myofibers. We suggest this mechanism resulting in a hybrid muscle fiber, primarily along the fiber tips, enables a smooth transition from muscle fiber characteristics towards tendon features essential for forming robust MTJs. We propose that dual characteristics of junctional cells could be a common mechanism for generating stable interactions between tissues throughout the musculoskeletal system.

## Abstract (web summary)

Classically, myogenic precursor cells derive from somites, and connective tissues derive from lateral plate mesoderm (LPM). Here the authors identify LPM derived fibroblasts that turn on a myogenic program and fuse to muscle fibers at muscle-tendon junctions, introducing fibroblast transcripts into myofibers.

## Introduction

Transplantation experiments in avian embryos as well as genetic investigations in mice performed over the last few decades identified the lineages, that make up the vertebrate limb musculoskeletal system. These have demonstrated that while myogenic precursor cells are somite-derived, the connective tissues, tendons, and bones are derived from the lateral plate mesoderm (LPM)^1–4^. However, single cell resolution techniques allow us to revisit these early observations and focus on specific regions, such as the sites of interaction between the distinct tissues to resolve their cellular contributions. One such focal point is the myotendinous junctions (MTJs).

Although being critical for muscle functioning in transmitting the force generated by the muscle to the tendons and skeletal elements, our understanding of the mechanisms that underlie MTJ development and maintenance are still relatively unclear. Importantly, while myofiber tips along the MTJs serve as the sites of interaction with the tendons, during embryonic and neonatal development when myofibers elongate via myoblasts’ fusion, the majority of fusion events also take place at these regions^5–9^. Accordingly multiple signaling pathways such as BMP and FGF signaling are tightly coordinated along the fiber tips^10,11^ although their exact contribution to fusion, myofiber elongation, and/or MTJ formation and maintenance is still unclear.

In this work, we examine the mechanisms underlying MTJ formation. We carry out a single cell transcriptome (scRNAseq) analysis of the MTJ region. This analysis reveals the presence of a unique cluster of cells expressing both myogenic as well as fibroblastic characteristics. Interestingly, cells in this cluster also express multiple genes known to be associated with MTJs. Surprisingly, this analysis further identifies that the secreted extracellular matrix (ECM) modifying enzyme Lysyl oxidase-Like 3 (LoxL3), an essential enzyme required for MTJ formation, that is secreted from myofiber tips^12^ is transcribed by fibroblasts and not by myogenic cells. Using fluorescent in situ hybridization (FISH) analyses and tracing of fibroblast nuclei in vitro and in vivo within the developing muscle, we identify a mechanism of horizontal RNA transfer by which LPM-derived fibroblasts transdifferentiate, switch on myogenic characteristics and fuse into the myofibers along the MTJs. We suggest recruitment of LPM-derived cells from muscle boundaries and their fusion into the myofibers is essential for normal MTJ development ensuring proper localization of proteins along these junctions.

## Results

### scRNAseq reveals cells with dual identities

**Fig. 1:**
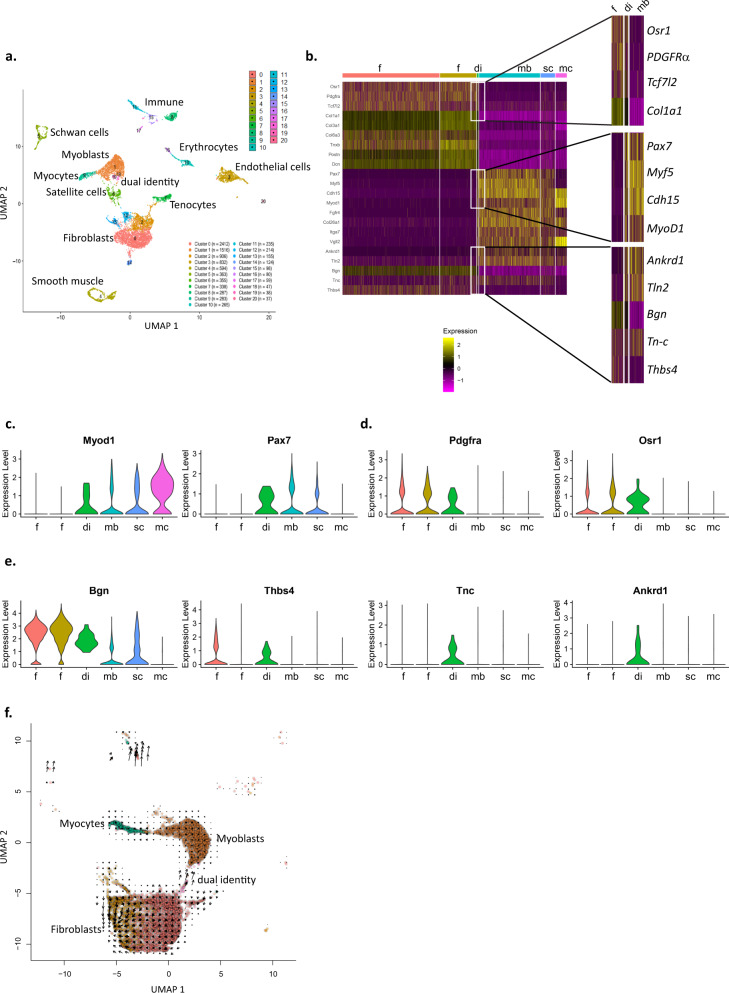
scRNA sequencing reveals cells with dual myogenic and interstitial transcriptional identities. UMAP depicting cell clusters (**a**) heat map (**b**) and violin plots of distinct genes associated with myogenesis (**c**) FAPs/fibroblasts (**d**), and tendon and MTJs (**e**). UMAP of RNA velocity analysis of myogenic (1,6,9), fibrogenic (0,2) and dual identity clusters (**f**). f fibroblasts, mb myoblasts, mc myocytes, sc satellite cells, di dual identity.

To investigate the cellular contributions to the MTJs, we carried out a single cell transcriptome analysis of the muscle-tendon region at P0 (https://www.ncbi.nlm.nih.gov/geo/query/acc.cgi?acc=GSE168153). At this stage, MTJs have already formed yet myoblast fusion at this region is high. Single cells (i.e., without contribution of syncytial myofibers) were used for the analysis. Transcriptome analysis identified 21 independent clusters (Fig. 1a). Clusters expressing the pan muscle connective tissue interstitial marker *PDGFRα* (marked as fibroblasts in the UMAP; Fig. 1a) and *Osr1* were identified. These included muscle connective tissue fibroblasts with high *Tcf4* (*Tcf7l2*) or fibro-adipogenic precursors (FAPs) expressing *ScaI* (*Ly6a*). These fibrogenic clusters expressed significantly distinct transcriptomes from those enriched for myogenic markers (consisting of myoblasts progenitors and *Myogenin* expressing myocytes; Supp. Fig. 1a). We could further identify tenocytes, endothelial, and mural cells (Supp. Fig. 1b–d). A small number of cells co-expressing myogenic and fibrogenic markers were identified in multiple clusters. Notably, while generally the above clusters could be associated with a single identity, a small number of cells clustered together (*n* = 38; dual identity cells) expressing both fibrogenic identity markers as *PDGFRα, Osr1, Col1a1, and Dcn* but also myogenic markers such as *Pax7*, *Myf5*, *MyoD1*, *M-Cadherin* (*Cdh15*) and others (Fig. 1b–e and Supp. Fig. 1a–g). Interestingly, cells in this cluster also express multiple MTJ and tenogenic markers such as *Ankrd1, Thbs4, Bgn, and Tn-C*, respectively^13–16^. Notably, some of these markers are specific to this cluster suggesting these dual identity cells form an independent cell cluster (Fig. 1e).

To further characterize the dual identity cells, we used Ingenuity Pathway Analysis (IPA) software. When comparing transcriptomes of this cluster to that of the myogenic clusters using the upstream regulator analysis tool, we find TGFβ, previously associated with MTJ formation^17^, being the most highly upregulated pathway in this cluster (*Z*-score 5.528, *p* value 1 × 10^−37^). This analysis further suggests BMP signaling, known to be highly activated along myofiber tips^11^(and Esteves de Lima, co-submitted), is activated in the dual identity cells (*Z*-score 3.544, *p* value 5.7 × 10^−7^). TGFβ and BMP signaling were also suggested to be activated when comparing these unique progenitors to fibrogenic clusters although to a lower extent (*Z*-score 1.69, *p* value 3 × 10^−10^; *Z*-score 2.13, *p* value 2.95 × 10^−3^, respectively), but not to the myogenic clusters. Altogether these results suggest these two cascades are significantly activated in the cluster containing the dual identity cells.

The dual identity cells expressed genes associated with myogenic proliferation (e.g., *Pax7* and *Myf5*) and early differentiation (e.g., *MyoD1* but not *Myogenin*). Accordingly, IPA analysis suggests they are much more motile and proliferative. WEB-based Gene Set Analysis Toolkit (WebGestalt^18^) using the differentially expressed genes (DEGs) in this cluster versus those of the myogenic or fibrogenic clusters was further carried out. Changes in ECM composition of the dual identity clustered cells only vs. myogenic clusters were highlighted. These differences further demonstrate that cells in this unique cluster express high levels of ECM genes more similar to that observed in fibrogenic and tendon cells reinforcing the notion these cells form a transition between the myofiber and the tendon within the MTJ.

Remarkably, RNA velocity, an analysis which predicts future cell fate based on abundance of nascent (unspliced) and mature (spliced) mRNA forms in single cell data^19^ suggests that the progenitors with a dual identity stem from fibrogenic clusters moving towards the myogenic identity (Fig. 1f and Supp. Fig. 1h–i). Hence raising the possibility that these progenitors transdifferentiate from fibroblasts into myogenic progenitors.

### *LoxL3* RNA is expressed by muscle interstitial cells

To begin to test the transdifferentiation idea, we probed a system where we previously demonstrated that the ECM modifying enzyme LoxL3 plays an essential role in MTJ development. In *LoxL3* mutant embryos, myofibers do not properly anchor along the MTJs^12^ (Supp. Fig. 2a–c). The scRNAseq analysis revealed *LoxL3* transcripts are primarily expressed in fibrogenic clusters and not in the myogenic ones (Supp. Fig. 1j). Surprisingly, this expression pattern contrasts with the expression of LoxL3 protein, which is expressed inside the myofiber tips (Supp. Fig. 2d–k) and being a secreted ECM-modifying enzyme is presumably secreted from their tips to modify the MTJ matrix promoting myofiber anchorage along the MTJ.

**Fig. 2:**
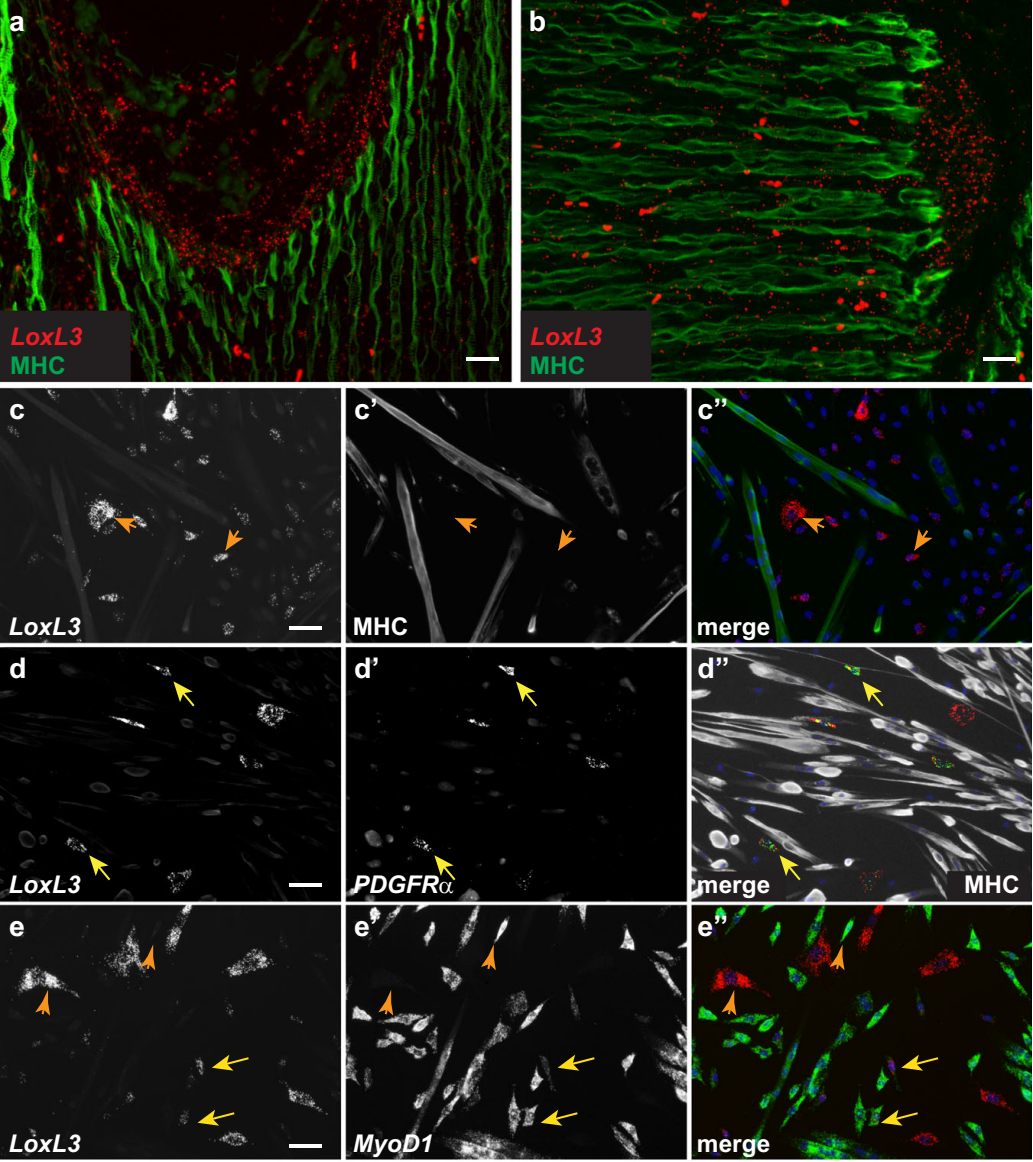
*LoxL3* RNA is expressed in muscle interstitial cells. FISH staining for *LoxL3* RNA (red) and MHC immunostaining (green) at E15.5 demonstrates LoxL3 is expressed close to myofiber tips yet primarily outside of the myofibers (**a**, **b**). FISH for *LoxL3* (**c**) or also for *PDGFRα* (**d**) and anti-MHC staining demonstrates that *LoxL3* RNA is not expressed in myofibers but in *PDGFRα* expressing interstitial cells. FISH for *LoxL3* and *MyoD1* (**e**) reveals that most cells express either one of these markers (orange arrows), some express both (yellow arrows). Images shown are representative of at least three independent embryos or cell isolation experiments. Scale bars = 50 µm.

To verify the scRNAseq results that suggest *LoxL3* RNA is expressed primarily by fibroblasts we carried out FISH analysis on tissue sections. In accordance with the above RNA sequencing results and in contrast to LoxL3 protein expression, we find *LoxL3* RNA is mostly expressed adjacent to the muscle tips yet primarily outside of the myofibers (Fig. 2a, b).

This unexpected observation of different protein and RNA expression lead us to further explore *LoxL3* RNA localization in a more controlled environment. We took advantage of our previous observations demonstrating that in in vitro differentiated myotubes, LoxL3 protein localization is maintained at the tips^12^. We therefore monitored *LoxL3* RNA in primary cultures containing myoblasts, myotubes (marked by myosin heavy chain, MHC) and interstitial fibroblasts/FAPs (marked by *PDGFRα*, Supp. Fig. 2l) (Fig. 2c–e). In accordance with the in vivo results, no *LoxL3* RNA expression was observed in myotubes (Fig. 2c-c″). In contrast, *LoxL3* mRNA was primarily observed in *PDGFRα* expressing fibroblasts/FAPs interstitial cells (Fig. 2d-d″). Staining for *LoxL3* and *MyoD1* RNAs revealed that most cells expressed either marker-myogenic cells expressed *MyoD1* whereas fibroblasts/FAPs expressed *LoxL3* (Fig. 2e-e″; orange arrowheads). Notably, ~33% of the *LoxL3* expressing cells also expressed *MyoD1* (Fig. 2e-e″; yellow arrows). Altogether these results demonstrate that in contrast to its protein expression, *LoxL3* RNA is primarily expressed by bona fide MCT fibroblasts/FAPs some of which also express *MyoD1*.

### LPM-derived fibroblasts fuse to growing myofibers

The observation that *LoxL3* and *PDGFRα* expressing cells could also express myogenic markers (Fig. 2e), together with the results of the RNA velocity analysis (Fig. 1f), led us to test whether these cells are LPM-derived fibroblasts that have switched on a myogenic program. Towards testing this hypothesis, we took advantage of the *Prx1*^*Cre*^ ^20^, a LPM-specific Cre driver line driven by a *Prrx1* limb enhancer^21^ previously suggested not to be expressed in myogenic cells^20,22,23^. scRNAseq confirmed *Prrx1* is expressed by fibrogenic and not myogenic cells. Notably this analysis further demonstrated *Prrx1* is also expressed by the dual identity cells (Supp. Fig. 3a) further reinforcing the above observations (Fig. 1f) that these cells are derived from the LPM.

To further test the validity of this Cre line in vivo, we crossed it to the *Rosa*^*nT-nG*^ reporter line^24^. In this reporter, all nuclei express tdTomato however upon Cre activity nuclear EGFP is switched on instead. Hence it enables the identification of LPM-derived nuclei in syncytial myofibers. Section immunostaining for Laminin marking the myofibers of E18.5 embryonic limb muscles demonstrates that no LPM-derived nuclei (EGFP-expressing) are found within the fibers away from the MTJ (Supp. Fig. 3b,b′). Along these lines, immunostaining for Pax3 marking myogenic precursors at E10.0 show no overlap with the LPM-derived EGFP expressing cells (Supp. Fig. 3c).

**Fig. 3:**
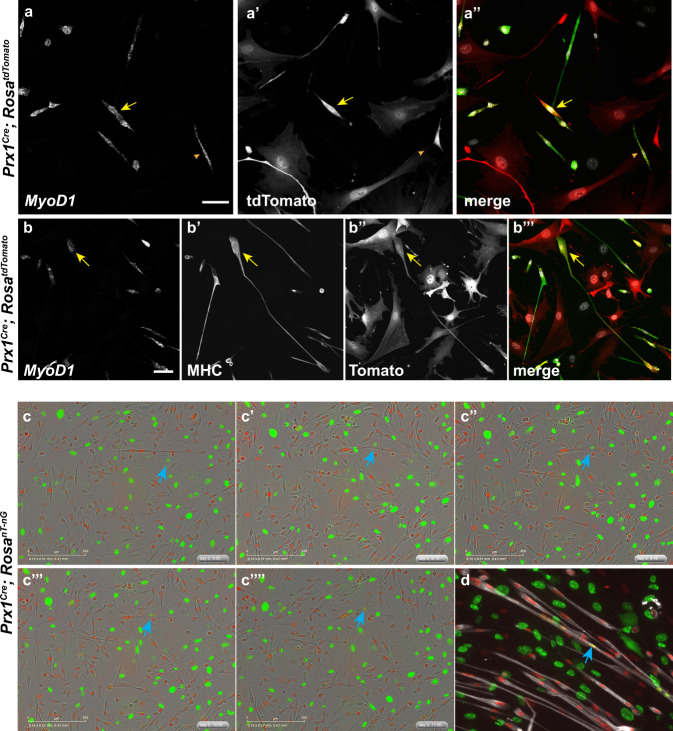
Cultured muscle interstitial cells fuse into growing myotubes. Primary culture of myoblasts and interstitial cells derived from *Prx1*^*Cre*^; *Rosa*^*tdTomato*^ following 24 h of differentiation demonstrates the presence of tdTomato expressing *MyoD1* (yellow) and MHC (green) expressing myofibers (**a**, **b**). Snapshots of live-cell imaging of *Prx1*^*Cre*^; *Rosa*^*nt-ng*^-derived primary culture after 48 h in differentiation media revealing fusion of fibroblast (blue arrow) into growing myofiber (**c**-**c**″“). Following 72 h in differentiation media, fibers marked by MHC (gray) with multiple fusion events are observed (**d** blue arrows). Shown are representative images of FISH and immunostaining results of at least three independent cell isolation experiments from the distinct genotypes. Scale bar in **a**, **b** = 50 µm and in **c** = 200 µm.

Our results above suggest that LPM-derived cells can transdifferntiate and switch on a myogenic program. To test whether this takes place, we crossed the *Prx1*^*Cre*^ to the *Rosa26R*^*tdTomato*^^ 25^, a cytoplasmic reporter, resulting in tdTomato expressing LPM-derived cells. FISH analysis was carried out on cultured cells derived from limb muscles. We find that *MyoD1* RNA is expressed also in a subset of LPM-derived fibroblasts (Fig. 3a-a″), and that tdTomato MHC expressing myotubes do form in these cultured cells (Fig. 3b-b″’).

The observation of tdTomato-expressing myotubes could be a consequence of several mechanisms that facilitate the introduction or transfer of the fibroblast-derived tdTomato RNA into the myofibers. Such mechanisms could include fusion of fibroblasts into the myofibers, extracellular vesicles released to the media that are then taken up by the myofibers or cellular extensions such as cytonemes between cell types^26–28^. The observation of adjacent *MyoD1* expressing cells, where one is tdTomato-positive and the other negative (Fig. 3a; yellow arrow) suggested this is not a random event and may result from a regulated process such as cell fusion, an event that is essential for myofiber growth. To directly test whether fibroblasts fuse into growing fibers, we crossed the *Rosa*^*nt-ng*^ reporter line that enables tracking of the distinct nuclei even in syncytia such as myofibers. Single cells were isolated from limb muscles of *Prx1*^*Cre*^; *Rosa*^*nt-ng*^ P0 neonates and cultured in low serum to facilitate myogenic differentiation. In these cultures, all myogenic cells’ nuclei are tdTomato-expressing while all LPM-derived fibroblasts/FAPs’ nuclei express EGFP. Culturing of sorted tdTomato-only or EGFP-only cells (Supp. Fig. 3d) demonstrates that, as expected, late or random Cre activity (and hence loss of tdTomato and gain of EGFP) does not occur in these culture conditions (Supp. Fig. 3e–g″). Live cell imaging confirms EGFP expressing fibroblasts/FAPs fuse into growing myofibers (Fig. 3c-c″“; blue arrow and Supp. Movie1). Following 72 h of in vitro differentiation ~7% of MHC expressing myotubes (*n* = 276) harbor at least one EGFP expressing nucleus derived from such a fusion event (Fig. 3d).

**Fig. 4:**
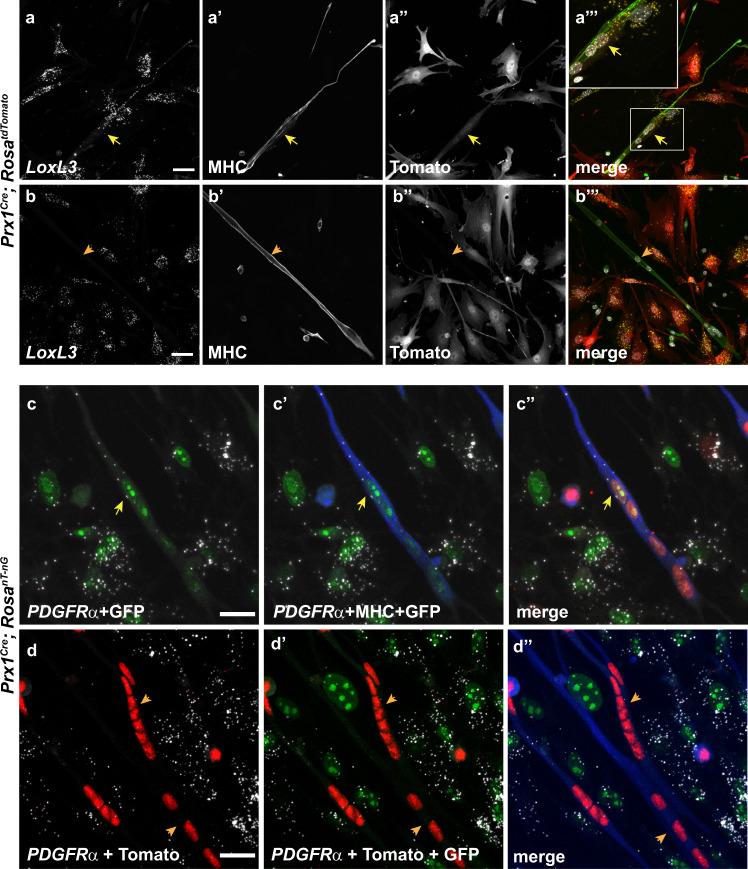
Fused LPM-derived cells actively transcribe and contribute fibroblast-associated genes to growing myotubes. FISH for *LoxL3* (**a**–**b**″′) or *PDGFRα* (**c**–**d**″) on primary cultured myoblasts and interstitial cells derived from *Prx1*^*Cre*^; *Rosa*^*tdTomato*^ (**a**, **b**) or *Prx1*^*Cre*^; *Rosa*^*nt-ng*^ (**c**, **d**) following 48 h of differentiation demonstrates that only in fibers where LPM-derived cells have fused (yellow arrows) but not in tdTomato-negative (**b-b**″′) or GFP-negative (**d-d**″) are *LoxL3* (**a**) or *PDGFRα* (**c**) RNA are observed. MHC in gray or green (**a**, **b**) or blue (**c**, **d**). Shown are representative images of FISH and immunostaining results of at least three independent cell isolation experiments from the distinct genotypes. Scale bar = 50 µm.

That LPM-derived interstitial cells can fuse into growing myotubes raised the possibility that fibroblast-specific RNAs would be transferred along, thus facilitating the expression of fibroblast/FAP-associated genes such as *LoxL3* and *PDGFRα* within the myotube. Accordingly, we find that only in tdTomato-positive fibers (i.e., have a contribution of at least one fibroblast) the fibroblast/FAP-specific RNAs of *LoxL3* and *PDGFRα* are found (Fig. 4a, b and Supp. Fig. 4). To test whether the LPM-derived cells that have fused into the myotubes instruct other nuclei to transcribe the fibroblast/FAP-specific RNAs or whether they are the ones that actively transcribe it, we monitored the expression of *PDGFRα* in myotubes derived from *Prx1*^*Cre*^*; Rosa*^*nt-ng*^ mice. We find that active transcription as monitored by two fluorescent foci inside the nucleus, occurs only in the EGFP-positive nuclei (Fig. 4c, d″). Overall, these results reveal that not only do LPM-derived interstitial cells fuse into growing fibers but that they also maintain, at least to some extent, their initial identities’ transcriptional program.

**Fig. 5:**
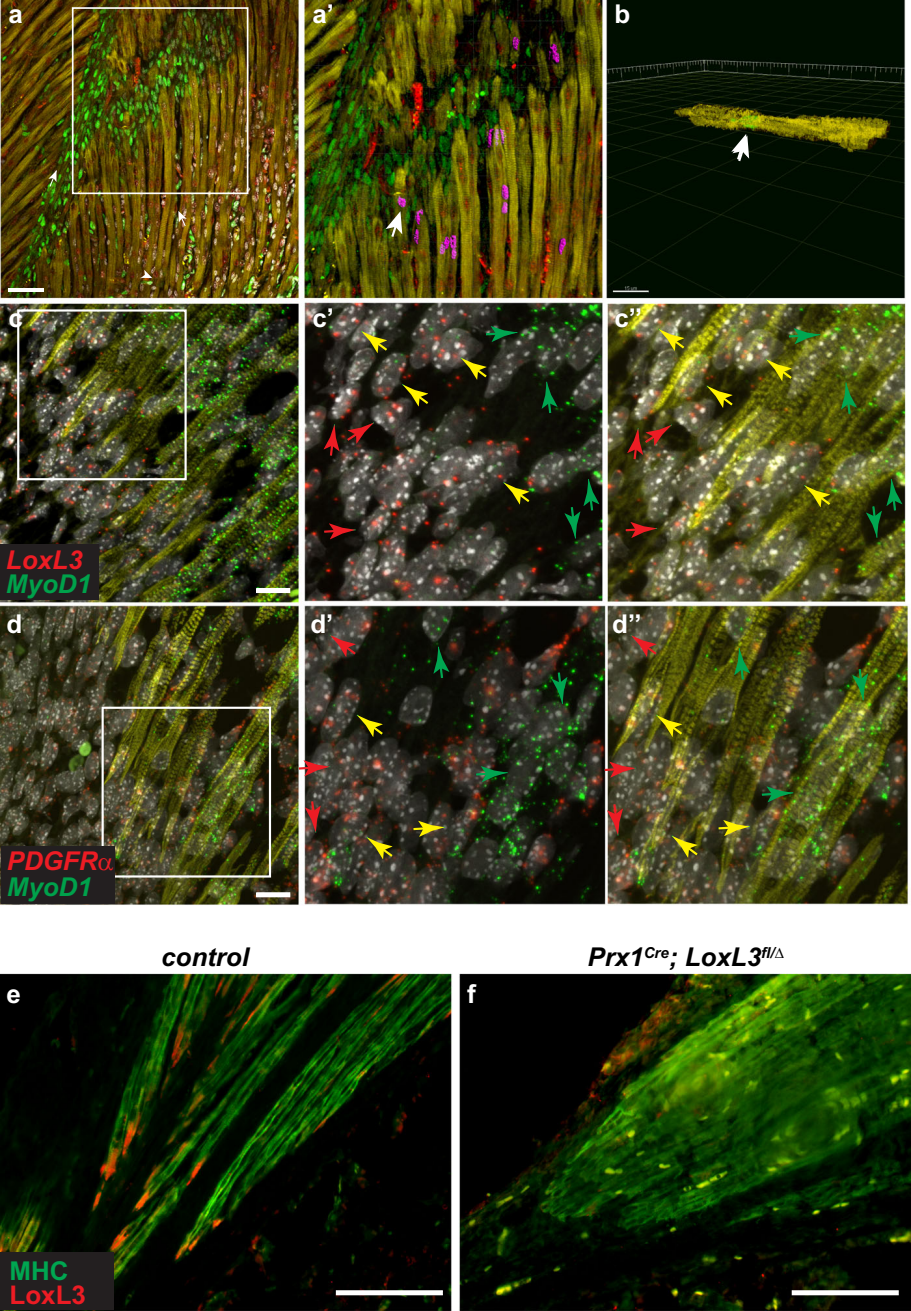
LPM-derived cells fuse into myofibers along the MTJ. P0 *Prx1*^*Cre*^; *Rosa*^*nt-ng*^ limbs were subjected to immunostaining for MHC (yellow), EGFP (green) and tdTomato (red) (**a**) followed by IMARIS analysis demonstrates LPM-derived cells fuse into fibers at the proximity of the MTJ (**a**′ purple; **b** lateral view of fiber section marked by white arrow in **a**′) (**a**, **b**). FISH for *LoxL3* and *MyoD1* (**c-c**″) or *PDGFRα* and *MyoD1* (**d-d**″) on E15.5 limb sections. Tagged images show magnification of boxed areas in **c**, **d**. Staining demonstrates spatial localization of the distinct nuclei at the fiber tips those located towards the center of the muscle express only *MyoD1* (green arrows in **c**′, **d**′), those closer to the tips (but also outside) express *MyoD1* and fibroblastic markers (yellow arrows in **c**′, **d**′) and those outside of the fiber towards the tendon express only fibroblastic markers (red arrows in **c**′, **d**′). Immunostaining for MHC (green) and LoxL3 (red) on E17.5 *control* (**e**) and *Prx1*^*Cre*^*LoxL3*^*fl/Δ*^ (**f**) demonstrates LoxL3 is missing at myofiber tips following its deletion in fibroblasts. IMARIS analyses (**a**–**b**), immunostaining and FISH results (**c**–**f**) are representative results derived from at least three independent embryos from distinct litters of the specific genotypes. Scale bar in **a**, **e**, **f** = 50 µm, in **b** = 15 µm and in **c**, **d** = 10 µm.

A series of experiments marking proliferating cells, have demonstrated that within the elongating muscles, progenitors fuse to elongating myofibers primarily along the tips adjacent to the MTJs^5–7^. Although the previous experiments did not monitor the identity of these progenitors, *Prx1*^*Cre*^*; Rosa*^*nt-ng*^ enable dissection of whether some of the fused cells are LPM-derived fibroblasts. Analysis of *Prx1*^*Cre*^*; Rosa*^*nt-ng*^ mice demonstrate that, as expected, the myofibers’ nuclei are primarily tdTomato-positive while EGFP expressing nuclei are found along the MTJs and in between myofibers (Fig. 5a). Should fibroblasts fuse into the growing myofibers also in vivo, we would expect that in proximity to the myofiber tips, adjacent to the MTJs, EGFP-expressing nuclei will be found. IMARIS 3D analysis of confocal images derived from wholemount immunostaining of muscles, demonstrates this is the case and the EGFP nuclei are primarily located close to the MTJs (Fig. 5a, b and Supp. Fig. 5a-a′ and Supp. Movies 2–3). Late activation of *Prx1*^*Cre*^ also in myogenic cells could, in principle, lead to EGFP expressing nuclei within the myofibers. However, should this be the case, then we would expect these EGFP nuclei to be present throughout the myofibers. Our cell culture analyses (Supp. Fig. 3e–g″) as well as our in vivo stainings which show that even in late stages of embryogenesis EGFP expressing nuclei are not present throughout the myofibers (Supp. Fig. 3) but only along the MTJs, suggests such late activation does not occur. Altogether these results demonstrate that fibroblast fusion into myofibers occurs as part of the normal developmental program, and confirm in vivo the in vitro observations of the dual origin of cells that make up the myofiber.

Fusion between myoblasts has been shown to be dependent on the transmembrane fusogene *myomaker* (*Mymk*)^29^. To test whether the LPM-derived cells fuse into the myofiber in a *Mymk*-dependent manner we deleted *Mymk* in the LPM lineage using the *Prx1*^*Cre*^, however no MTJ abnormalities were observed (Supp. Fig. 5b, c). Overall, these observations suggest that the fusion of LPM-derived cells into the myofiber is dependent on a mechanism not involving *Mymk* in fibroblasts and potentially consistent with the lack of need for *Mymk* in myofibers for fusion^30,31^.

The finding that LPM-derived fibroblasts fuse into the myofiber in proximity to the MTJs led us to test, whether *LoxL3*-expressing and *PDGFRα*-expressing nuclei can be found in vivo within myofibers adjacent to the tips. FISH data for these fibroblast markers and for *MyoD1*, establishes that adjacent to the myofiber tips, both within them but also outside, nuclei actively transcribing fibroblastic markers and *MyoD1* are present [Fig. 5c, d (yellow arrows in c′ and d′); Supp. Fig. 5d–f″′]. Interestingly, the nuclei located towards the center of the fiber express only *MyoD1* (green arrow in Fig. 5c′, d′) whereas those located farther away and outside of the fiber express only the fibroblastic marker (red arrow in Fig. 5c′, d′). Since some of the nuclei expressing both myogenic and fibrogenic markers are located at the tip of the fiber, where MHC expression is sometimes less prominent, we were not able to conclusively quantify the number of these nuclei that are fully contained within the fiber (Supp. Fig. 5g).

In accordance with the above results, *LoxL3* deletion in the LPM-derived cells using the *Prx1*^*Cre*^ deleter line but not in the myogenic progenitors using the *Pax3*^*Cre*^ line (not shown), results in MTJ defects (Supp. Fig. 5h, i) and in loss of LoxL3 protein expression from the myofiber tips (Fig. 5e, f). Overall, these results demonstrate that the myofiber tips at the MTJ region encompass unique nuclei that are of LPM-origin that also express fibroblastic markers, and that this hybrid nature of the muscle fiber is critical for formation and maintenance of the MTJs.

## Discussion

Here we explored the mechanisms underlying the building of the musculoskeletal system focusing on the MTJs. Single cell resolution techniques allowed us to revisit the paradigms depicting muscle development. We identified that LPM-derived fibroblasts transdifferentiate by switching on the myogenic program and fuse into the myofiber. We demonstrate this fusion is part of the normal developmental program generating syncytial myofibers with hybrid developmental origins. Our results suggest this process is essential for MTJ formation. Notably, in an accompanying paper D. Duprez and colleagues (Esteves de Lima, co-submitted) find this dual origin contribution to the myofibers at the MTJ occurs also in avian development. Reinforcing our observations, a unique group of nuclei expressing myogenic and fibrogenic genes (MTJ-B), the majority of which are shared by the dual identity cells, located at the MTJs, was recently identified in single nuclei sequencing of adult and regenerating myofibers^32^ further suggesting the hybrid nature of muscle fibers.

FISH analysis demonstrated *LoxL3* is transcribed by fibroblasts. Accordingly, upon its deletion in the LPM-derived cells we observed LoxL3 protein loss. Interestingly, LoxL3 protein, and hence its loss, was observed in the myofibers. Notably, while myofiber nuclei are thought to be somite-derived, the rest of the limb’s tissue, including the tendons and muscle connective tissues are derived from the LPM. Using live imaging and lineage tracing, we identified that LPM-derived fibroblasts can fuse into the elongating myofibers. Upon fusion, these fibroblasts maintain, at least partially, their original transcriptional program and thus facilitate the expression of fibroblast-specific genes in myofibers altogether leading to localized expression of fibroblast-specific genes in myofibers. We have previously demonstrated that LoxL3 oxidizes fibronectin along the myofiber tips. This oxidation promotes rapid activation of the integrin receptors expressed on the myofibers’ termini^12^. Hence proper localization of LoxL3 enzymatic activity along the myofibers tips is critical for the fibers’ adhesion along the forming MTJ. Thus, as a by-product of the LPM-derived cells’ fusion to the myofiber tips, LoxL3 becomes localized to their termini and can then act locally along the MTJ.

Recent work has demonstrated the tendon-bone junctions is also composed of two cell types: tendon fibroblasts and chondrocytes^33,34^. Interestingly, this junction is dependent on TGFβ and BMP4 signaling^17,33,34^. Notably, tendon development is highly dependent on TGFβ signaling^35,36^ and BMP4 activity is prominent in muscle fiber tips^11^. Our results also point to an involvement of these two pathways in the dual identity cells that contribute to the MTJs putting forward the possibility that cells with dual programs are required for generating junctions between tissues.

## Methods

### Mice

All experiments involving mice conform to the relevant regulatory standards (Technion IACUC and national animal welfare laws, guidelines and policies). All mice are housed in IVC’s (Techniplast) according to space requirements defined by the NRC. All rooms are set to have 22 °C ± 2° and humidity of 30–70%. All HVAC parameters are controlled by a central computerized monitoring system. Light cycle is set to full light 10 h half-light 2 h and complete darkness 12 h, light cycle is monitored by the computerized central system. Embryonic day (E) was staged according to Kaufmann^37^; noon of the day a vaginal plug was observed was marked as E0.5.

The *LoxL3* allele and the *Prx1*^*Cre*^ were previously described^12,38^, respectively. *Rosa26R*^*tdTomato*^ (also known as Ai9)^25^ and *Rosa26*^*nTnG*^ were purchased from JAX mice. All mice are kept and bred on a C57Bl/6 background purchased from Envigo (https://www.envigo.com).

### Mouse genotyping

Mice were PCR genotyped using the following primers:

*LoxL3*: LoxL3-5armWTF: GCCAGGGTGAAGTGAAAGAC; LoxL3-CritWTR: GATCTGGGATGCTGAAGACC; Tm1a-5mut-R1: GAACTTCGGAATAGGAACTTCG.

300 and 100 bp represent wild-type and mutant PCR products, respectively.

Cre: CreORF REV: ATCCAGGTTACGGATATAGT; CreORF FWD: ATCCGAAAAGAAAACGTTGA. 500 bp represent a cre positive PCR product.

nTnG: nT-nG_1: CCAGGCGGGCCATTTACCGTAAG; nT-nG_2: GGAGCGGGAGAAATGGATATG; nTnG 3: AAAGTCGCTCTGAGTTGTTAT. 603 and 320 bp represent wild-type and homozygous presence of nTnG PCR products, respectively.

tdTomato: RosaTomato WT_FWD: AAGGGAGCTGCAGTGGAGTA; RosaTomato WT_REV: CCGAAAATCTGTGGGAAGTC. Fragment size of 196 bp represent the tomato transgene, while a fragment size of 297 bp represent the wild- type.

The complete set of primers used is shown in Supp. Table 1.

### Fluorescent in situ hybridization (FISH)

FISH was carried out using the RNAScope system according to the manufacturers’ protocol^39^, on FFPE forelimb and hindlimb sections and also in primary cell cultures. Briefly, the sections were baked in a dry oven for 1 h at 60 °C, deparaffinized using xylene and ethanol, air dried and incubated in hydrogen peroxide for 10 min. For the antigen retrieval step, slides were boiled in an anti-retrieval solution for 2 min, transferred to ethanol for 3 min and air dried. Slides were then incubated with an RNAScope special protease for 30 min. Designed target probes of *LoxL3* (431359; RNAScope), *MyoD1* (316081-C2; RNAScope), or *PDGFRα* (480661-C3; RNAScope) were added to the slides and incubated for 2 h at 40 °C. A series of probe amplifiers were added afterwards. At the end of the protocol, after adding the fluorophores, immunofluorescent staining was carried out. For cultured adherent cells, a similar protocol was carried out with the exception that the hydrogen peroxide incubation commenced directly after fixation (4% PFA).

All assays were repeated at least at three time points using different cell cultures or tissue sections from distinct embryos/mice.

### Immunohistochemistry

Section and whole-mount immunohistochemistry were performed essentially as previously described^40^. Briefly, 5 µm paraffin or 10 µm OCT embedded tissue sections were incubated over-night at 4 °C with the following antibodies at the concentrations below. Antigen retrieval was performed only when LoxL3 antibody was used. Wholemount immunostaining was carried out essentially with the same primary antibody concentrations although incubation periods were extended to 2 weeks in 4C (for both primary and secondary antibodies). The following antibodies were used: anti-Myosin (A4.1025, 1:300; DSHB); anti-GFP (A6455, 1:500; Invitrogen); anti-RFP (5F8, 1:500; Chromotek); anti-LOXL3^12^ (1:100).

All assays were repeated at least at three time points using different cell cultures or tissue sections from distinct embryos/mice.

### Fluorescent cell sorting

*Prx1*^*Cre*^; *Rosa26*^*nTnG*^ P0 neonate limb muscles were sliced into pieces, incubated with collagenase for 1 h and then with trypsin for half an hour. Tissues were then centrifuged for 15 min at 317×*g*, resuspended in DMEM (containing 1% l-glutamine and 1% penicillin and streptomycin (PS) + 10% FCS, filtered, re-centrifuged, re-suspended in BIO-AMF-2 (Biological Industries) and plated on gelatin coated plates. Cells were then cultured for 24 h and then sorted using FACSAria™ IIIu (BD Biocsiences) and data analyzed using BD FACS Diva8.0.1 software. Gating strategy was the following: Cells were initially gated on FSC-A/SSC-A to exclude outliers and cell debris. Gated cells were then single gated twice based on FSC-W/FSC-H and then by SSC-W/SSC-H. Single cells were then plotted on B530/30 (GFP) versus YG610/20 (RFP). Single RFP expressing cells and single GFP expressing cell were sorted and cultured.

### Generation of primary cultures

P0 neonate limb muscles were sliced into pieces, incubated with collagenase for 1 h and then with trypsin for half an hour. Tissues were then centrifuged for 15 min at 317×*g*, resuspended in DMEM (containing 1% l-glutamine and 1% penicillin and streptomycin (PS) + 10% FCS, filtered, re-centrifuged, re-suspended in BIO-AMF-2 (Biological Industries) and plated on gelatin coated plates. Differentiation was induced by incubating the cells in DMEM (containing 1% l-glutamine and 1% PS) + 4% horse serum for 3 days.

### 10× Genomics single cell general cell preparation

Single cell separation and library construction according to 10× protocol (Chromium Single Cell 3′ Library & Gel Bead Kit v2). Briefly, the MTJ region was dissected from P0 neonates and single cells were isolated (as described) and suspended with BIO-AMF-2 (Biological Industries). The cells were mixed thoroughly using a wide-bore pipette tip and counted. Cells were centrifuged at 317×*g* for 5 min at RT, suspended with 0.04% BSA PBS, re-centrifuged and then re-suspended with the appropriate volume of 0.04% BSA PBS to achieve a target cell concentration in the range of 700–1200 cell/µl. Following cell isolation, library construction was immediately carried out. Cell Ranger software (V3.0.2) was used for data QC and extraction of transcripts’ counts from raw data.

### Bioinformatic analysis

Seurat R package (Seurat_3.0.0.9100) was used for filtering, clustering and expression distribution of selected cluster-specific genes. SingleR R Package (SingleR_0.2.2) was used for unbiased cell type recognition of scRNA-seq. Cells with the following parameters were excluded: >8% mitochondrial UMI counts; less than 200 unique gene counts; over 4000 unique gene counts. Overall, 10,456 cells entered the analysis and 9238 cells were used for the bioinformatics analysis after filtration. In addition, genes detected in less than three cells were filtered out. WebGestalt (WEB-based Gene SeT AnaLysis Toolkit) a functional enrichment analysis web tool and Ingenuity Pathway Analysis (Qiagen) were used to identify specific pathways and processes within the data.

RNA velocity analysis was conducted as follows: A loom file from 10× output using velocyto.R with default parameters was used^19^. In summary estimating RNA Velocity using Seurat was created and imported to R and an RNA velocity analysis was conducted for the whole data. The cluster labels from the initial analysis were assigned to the velocity analysis in order to compare between the results. Cells that didn’t assign to any initial cluster were assigned as cluster “0”. RNA velocity was calculated on this data. Furthermore, cells that were labeled as fibroblasts, myoblasts, satellite cells, myocytes, and dual identity were extracted and analyzed using RNA velocity without re-clustering the data.

### Reporting summary

Further information on research design is available in the Nature Research Reporting Summary linked to this article.

## Supplementary information

Supplementary Information

Reporting Summary

Description of Additional Supplementary Files

Supplementary Movie 1

Supplementary Movie 2

Supplementary Movie 3

## Data Availability

Source data are provided with this paper. The datasets generated and/or analyzed during the current study are available in the GEO repository [https://www.ncbi.nlm.nih.gov/geo/query/acc.cgi?acc=GSE168153].
