# Supplementary material for: Fibroblast fusion to the muscle fiber regulates myotendinous junction formation
Source: Nat Commun. 2021 Jun 22;12:3852. doi: 10.1038/s41467-021-24159-9 (PMC8219707; doi:10.1038/s41467-021-24159-9)
Supplement: Supplementary file 2 — Reporting Summary [file 41467_2021_24159_MOESM2_ESM.pdf]

nature research

Corresponding author(s): Peleg Hasson

Last updated by author(s): May 8, 2021

Reporting Summary

Nature Research wishes to improve the reproducibility of the work that we publish. This form provides structure for consistency and transparency in reporting. For further information on Nature Research policies, see our Editorial Policies and the Editorial Policy Checklist.

Statistics

For all statistical analyses, confirm that the following items are present in the figure legend, table legend, main text, or Methods section.

n/a	Confirmed	
☒	☐	The exact sample size (*n*) for each experimental group/condition, given as a discrete number and unit of measurement
☐	☒	A statement on whether measurements were taken from distinct samples or whether the same sample was measured repeatedly
☒	☐	The statistical test(s) used AND whether they are one- or two-sided<br>*Only common tests should be described solely by name; describe more complex techniques in the Methods section.*
☒	☐	A description of all covariates tested
☒	☐	A description of any assumptions or corrections, such as tests of normality and adjustment for multiple comparisons
☒	☐	A full description of the statistical parameters including central tendency (e.g. means) or other basic estimates (e.g. regression coefficient) AND variation (e.g. standard deviation) or associated estimates of uncertainty (e.g. confidence intervals)
☒	☐	For null hypothesis testing, the test statistic (e.g. *F*, *t*, *r*) with confidence intervals, effect sizes, degrees of freedom and *P* value noted<br>*Give P values as exact values whenever suitable.*
☒	☐	For Bayesian analysis, information on the choice of priors and Markov chain Monte Carlo settings
☒	☐	For hierarchical and complex designs, identification of the appropriate level for tests and full reporting of outcomes
☒	☐	Estimates of effect sizes (e.g. Cohen's *d*, Pearson's *r*), indicating how they were calculated

Our web collection on statistics for biologists contains articles on many of the points above.

Software and code

Policy information about availability of computer code

Data collection	Single cell separation and library construction according to 10x protocol (Chromium Single Cell 3' Library & Gel Bead Kit v2). Briefly, the MTJ region was dissected from P0 neonates and single cells were isolated (as described) and suspended with BIO-AMF-2 (Biological Industries). The cells were mixed thoroughly using a wide-bore pipette tip and counted. Cells were centrifuged at 1300 rpm for 5 min at RT, suspended with 0.04% BSA PBS, re-centrifuged and then re-suspended with the appropriate volume of 0.04% BSA PBS to achieve a target cell concentration in the range of 700-1200 cell/ul. Following cell isolation, library construction was immediately carried out. Cell Ranger software (V3.0.2) was used for data QC and extraction of transcripts' counts from raw data.
Data analysis	Seurat R (Seurat_3.0.0.9100) package was used for filtering, clustering and expression distribution of selected cluster-specific genes. SingleR R Package (SingleR_0.2.2) was used for unbiased cell type recognition of scRNA-seq. Cells with the following parameters were excluded: >8% mitochondrial UMI counts; less than 200 unique gene counts; over 4,000 unique gene counts. Overall, 10,456 cells entered the analysis and 9,238 cells were used for the bioinformatics analysis after filtration. In addition, genes detected in less than 3 cells were filtered out. WebGestalt (WEB-based Gene SeT AnaLysis Toolkit) a functional enrichment analysis web tool and Ingenuity Pathway Analysis (Qiagen) were used to identify specific pathways and processes within the data.<br>RNA velocity analysis was conducted as follows: A loom file from 10x output using velocyto.R with default parameters was used. In summary estimating RNA Velocity using Seurat was created and imported to R and an RNA velocity analysis was conducted for the whole data. The cluster labels from the initial analysis were assigned to the velocity analysis in order to compare between the results. Cells that didn't assign to any initial cluster were assigned as cluster "0". RNA velocity was calculated on this data. Furthermore, cells that were labeled as fibroblasts, myoblasts, satellite cells, myocytes and dual identity were extracted and analyzed using RNA velocity without re-clustering the data.

For manuscripts utilizing custom algorithms or software that are central to the research but not yet described in published literature, software must be made available to editors and reviewers. We strongly encourage code deposition in a community repository (e.g. GitHub). See the Nature Research guidelines for submitting code & software for further information.

Data

Policy information about availability of data

All manuscripts must include a data availability statement. This statement should provide the following information, where applicable:

- Accession codes, unique identifiers, or web links for publicly available datasets
- A list of figures that have associated raw data
- A description of any restrictions on data availability

The datasets generated and analyzed during the current study are available in the GEO repository (Accession GSE168153ID)

Field-specific reporting

Please select the one below that is the best fit for your research. If you are not sure, read the appropriate sections before making your selection.

- [x] Life sciences
- [ ] Behavioural & social sciences
- [ ] Ecological, evolutionary & environmental sciences

For a reference copy of the document with all sections, see nature.com/documents/nr-reporting-summary-flat.pdf

Life sciences study design

All studies must disclose on these points even when the disclosure is negative.

Sample size	A minimum of three biological and technical repeats was used for each of the assays. In the case of wholemount or section immunostaining or FISH, embryos from three distinct litters were used in order to ascertain the obtained results.
Data exclusions	no data was excluded
Replication	Each assay was carried out at least three times at distinct time points using different cultures or embryos. All replicates were successful.
Randomization	Tissues or cells used per assay was used randomly and based on the genotype
Blinding	Data acquisition and image analyses (e.g., IMARIS) was carried out blindly. Once all data has been acquired and analyzed the data was then compiled into a an data set.

Reporting for specific materials, systems and methods

We require information from authors about some types of materials, experimental systems and methods used in many studies. Here, indicate whether each material, system or method listed is relevant to your study. If you are not sure if a list item applies to your research, read the appropriate section before selecting a response.

Materials & experimental systems

n/a	Involved in the study	
[ ]	[x]	Antibodies
[x]	[ ]	Eukaryotic cell lines
[x]	[ ]	Palaeontology and archaeology
[ ]	[x]	Animals and other organisms
[x]	[ ]	Human research participants
[x]	[ ]	Clinical data
[ ]	[ ]	Dual use research of concern

Methods

n/a	Involved in the study	
[x]	[ ]	ChIP-seq
[ ]	[x]	Flow cytometry
[x]	[ ]	MRI-based neuroimaging

Antibodies

Antibodies used	anti-Myosin (A4.1025, 1:300; DSHB); anti-Pax3 (Pax3; 1:100; DSHB); anti-GFP (A6455, 1:500; Invitrogen); anti-RFP (5F8, 1:500; Chromotek); anti-LOXL3 (1:100; previously reported by Kraft-Sheleg et al., Developmental Cell 36, 550–561, March 7, 2016). The LOXL3 polyclonal antibody was raised by ENCO Scientific Services by immunizing rabbits with a protein corresponding to aa 71-417 of the human protein. The antibody was kindly given to us by Prof. Gera Neufeld (Technion).
Validation	LoxL3 antibody validation was carried out on LoxL3 mutant embryos (described in the manuscript). No staining was observed in the mutant embryos. We have previously validated its specificity in western blot as described in Kraft-Sheleg et al., Developmental Cell 36, 550–561, March 7, 2016.<br>A4.1025 is described in Developmental Studies Hybridoma Bank's website (https://dshb.biology.uiowa.edu/A4-1025). It has been validated in western blot, ELISA and immunostaining and the validation steps are described in Hughes SM et al., Developmental Biology 158 (183-199) 1993.<br>According to the manufacturer's website, anti-GFP (A6455; Invitrogen) specificity was demonstrated by detection of different targets

	fused to GFP tag in transiently transfected lysates tested (https://www.thermofisher.com/antibody/product/GFP-Antibody-Polyclonal/A-6455).<br>Anti-RFP (5F8, Chromotek) is described in https://www.chromotek.com/products/detail/product-detail/rfp-antibody-5f8/<br>Anti-Pax3 is described in the DSHB website (https://dshb.biology.uiowa.edu/Pax3).

Animals and other organisms

Policy information about studies involving animals; ARRIVE guidelines recommended for reporting animal research

Laboratory animals	Mice used in this study were kept at the designated animal facility according to animal welfare laws. C57Bl/6 (males and females) mice were purchased from Envigo (https://www.envigo.com) and LoxL3 mice were bred in our facility. Breeding was carried out at ~8 weeks of age. Timed matings were used in cases where embryos were used.<br>When crossing the Rosa26:nTnG, Rosa26RtdTomato, Mymk flox or LoxL3 flox mice with the Prx1Cre, in all cases the Cre expressing mouse was the male and it was mated with ~8 weeks old females.<br>All mice are housed in IVC's(Techniplast) according to space requirements defined by the NRC. All rooms are set to have 22º C ± 2º and humidity of 30-70% . All HVAC parameters are controlled by a central computerized monitoring system. Light cycle is set to full light 10 h half-light 2h and complete darkness 12 h, light cycle is monitored by the computerized central system.
Wild animals	No wild-type animals were used in this study.
Field-collected samples	No field collected samples were used in the study.
Ethics oversight	All experiments involving mice conform to the relevant regulatory standards - Technion IACUC and national animal welfare laws, guidelines and policies.

Note that full information on the approval of the study protocol must also be provided in the manuscript.

Dual use research of concern

Policy information about dual use research of concern

Hazards

Could the accidental, deliberate or reckless misuse of agents or technologies generated in the work, or the application of information presented in the manuscript, pose a threat to:

No	Yes	
☒	☐	Public health
☒	☐	National security
☒	☐	Crops and/or livestock
☒	☐	Ecosystems
☒	☐	Any other significant area

Experiments of concern

Does the work involve any of these experiments of concern:

No	Yes	
☒	☐	Demonstrate how to render a vaccine ineffective
☒	☐	Confer resistance to therapeutically useful antibiotics or antiviral agents
☒	☐	Enhance the virulence of a pathogen or render a nonpathogen virulent
☒	☐	Increase transmissibility of a pathogen
☒	☐	Alter the host range of a pathogen
☒	☐	Enable evasion of diagnostic/detection modalities
☒	☐	Enable the weaponization of a biological agent or toxin
☒	☐	Any other potentially harmful combination of experiments and agents

Flow Cytometry

Plots

Confirm that:

☒ The axis labels state the marker and fluorochrome used (e.g. CD4-FITC).

☒ The axis scales are clearly visible. Include numbers along axes only for bottom left plot of group (a 'group' is an analysis of identical markers).

☒ All plots are contour plots with outliers or pseudocolor plots.

☒ A numerical value for number of cells or percentage (with statistics) is provided.

Methodology

Sample preparation	P0 neonate limb muscles were sliced into pieces, incubated with collagenase for 1 hour and then with trypsin for half an hour. Tissues were then centrifuged for 15 minutes at 1300 rpm, resuspended in DMEM (containing 1% L-glutamine and 1% penicillin and streptomycin (PS) + 10% FCS, filtered, re-centrifuged, re-suspended in BIO-AMF-2 (Biological Industries) and plated on gelatin coated plates. Cells were then cultured for 24 hours and then sorted.
Instrument	FACSAria™ IIIu (BD Biocsiences)
Software	BD FACS Diva8.0.1
Cell population abundance	31.0% of total cells were sorted for GFP only while 21.5% of total cells were sorted for tdTomato only.
Gating strategy	Cells were initially gated on FSC-A/SSC-A to exclude outliers and cell debris. Gated cells were then single gated twice base on FSC-W/FSC-H and then by SSC-W/SSC-H. Single cells were then plotted on B530/30 (GFP) versus YG610/20 (RFP). Single RFP expressing cell and Single GFP expressing cell were sorted for downstream application.

☒ Tick this box to confirm that a figure exemplifying the gating strategy is provided in the Supplementary Information.
