# Supplementary material for: Fibroblast fusion to the muscle fiber regulates myotendinous junction formation
Source: Nat Commun. 2021 Jun 22;12:3852. doi: 10.1038/s41467-021-24159-9 (PMC8219707; doi:10.1038/s41467-021-24159-9)
Supplement: Supplementary file 3 — Description of Additional Supplementary Files [file 41467_2021_24159_MOESM3_ESM.pdf]

Description of Additional Supplementary Files

Supplementary Movie 1. LPM-derived cells fuse into cultured myotubes. Live imaging of cultured cells derived from Prx1Cre; RosanTnG P0 limb muscles. EGFP expressing LPM-derived cells fuse into myotubes (Tomato positive). Yellow arrow marks a fibroblast that fuses with the forming myotube.

Supplementary Movie 2. LPM-derived cells fuse into myofibers in vivo in proximity to the MTJ. IMARIS 3D analysis of confocal images taken along the MTJ. Myoblasts are marked with tomato+ve nuclei, LPM derived cells are EGFP+ve and myofibers stained for myosin heavy chain (yellow). Movie focuses on one EGFP nuclei that is located adjacent to the MTJ.

Supplementary Movie 3. LPM-derived nuclei fuses with myofibers in vivo. High magnification of myofibers adjacent to the MTJ. Four nuclei are shown, one of them (EGFP+ve) is LPM-derived.
