# Supplementary material for: Fibroblast fusion to the muscle fiber regulates myotendinous junction formation
Source: Nat Commun. 2021 Jun 22;12:3852. doi: 10.1038/s41467-021-24159-9 (PMC8219707; doi:10.1038/s41467-021-24159-9)
Supplement: Supplementary file 1 — Supplementary Information [file 41467_2021_24159_MOESM1_ESM.pdf]

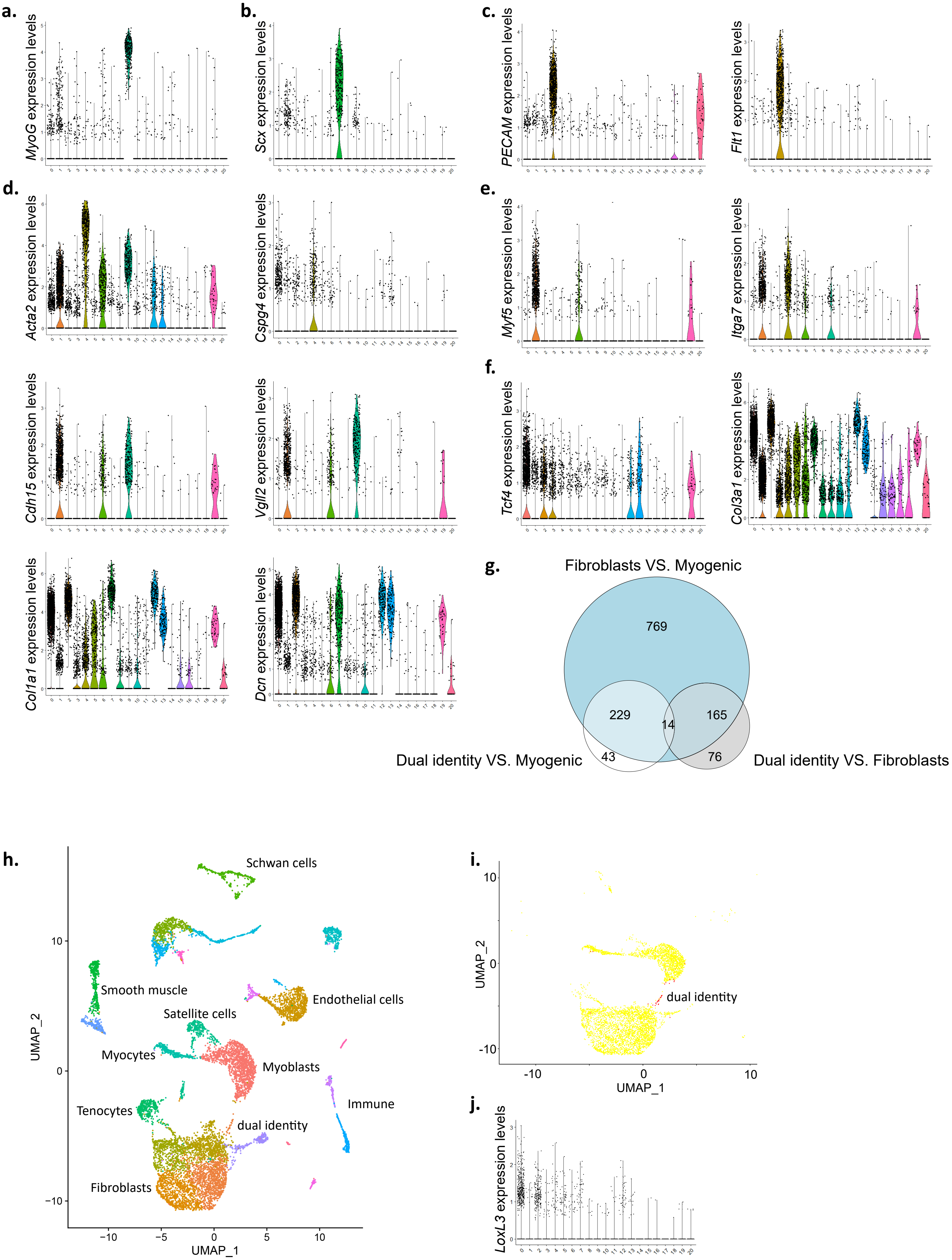

Supp. Figure 1

Supplementary Figure 1. ***scRNA seq highlights dual characteristics of cluster 19 cells.*** Violin plots depicting cell markers of myocytes (a), tenocytes (b), endothelial cells (c), smooth muscle cells (d) myogenic cells (e) and fibroblasts (f). Venn diagram depicting differentially expressed gene comparisons between myogenic and fibroblastic cell clusters and with dual identity cluster (g). UMAP plot derived from RNA velocity analysis of all clusters (h) and of myogenic (1,6,9), fibrogenic (0,2) and dual identity clusters. All dual identity cells are highlighted in red demonstrating the location of these cells as intermediates between fibroblasts/FAP clusters and myogenic ones (i). Violin plot of LoxL3 expression in distinct clusters (j).

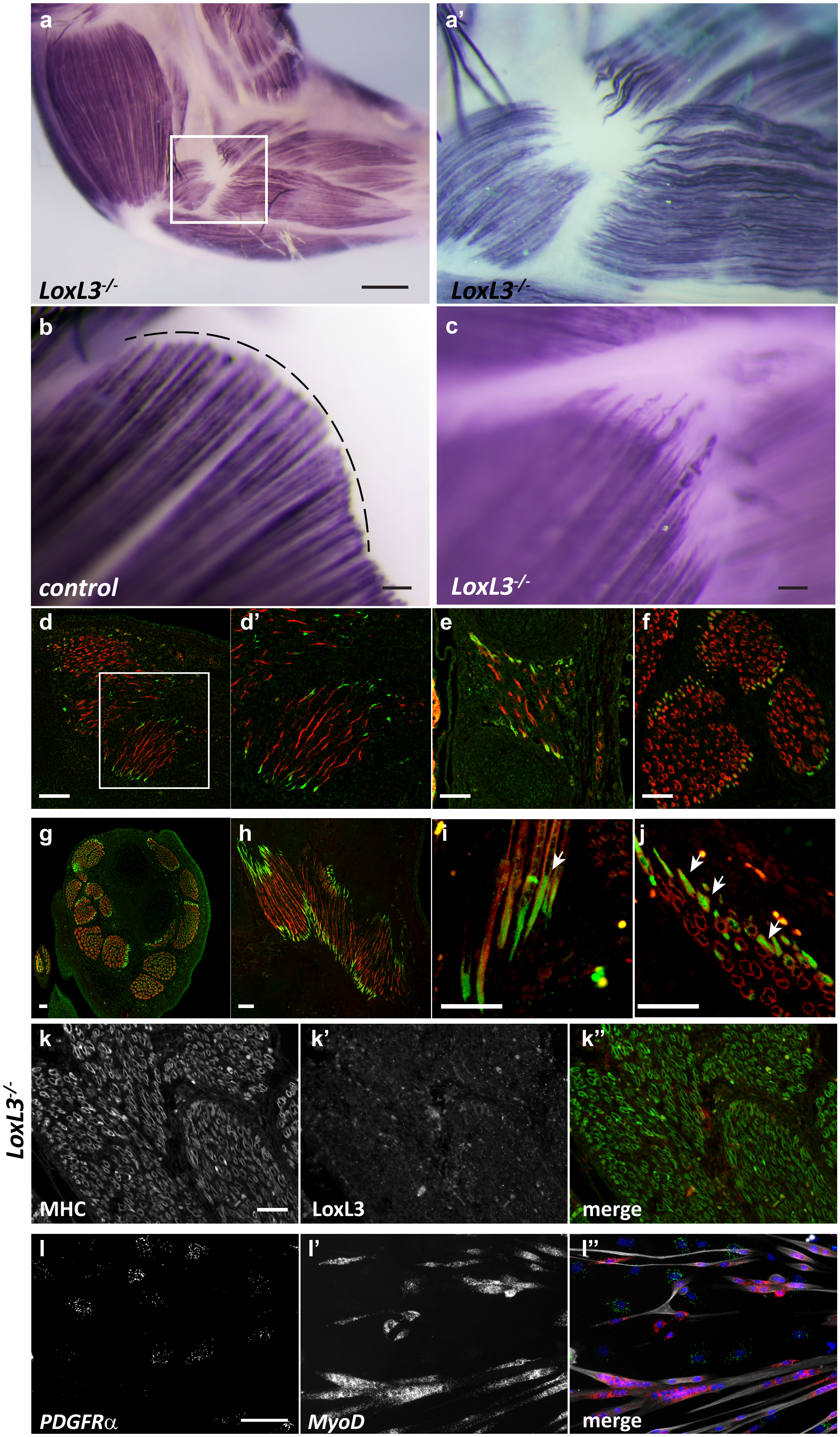

a
a’
$LoxL3^{-/-}$
$LoxL3^{-/-}$
b
c
control
$LoxL3^{-/-}$
d
d’
e
f
g
h
i
j
k
k’
k’’
$LoxL3^{-/-}$
MHC
LoxL3
merge
l
l’
l’’
PDGFRα
MyoD
merge

Supp. Fig. 2

Supplementary Figure 2. LoxL3 *expressed in myofiber tips is required for MTJs.* Wholemount MHC immunostaining on E18.5 forelimbs of LoxL3 mutant (a, a'), control (b) and LoxL3 mutant (c) embryos. Immunostaining for LoxL3 (green) and MHC (red) on E12.5 forelimb (d, d'), E13.5 rib (e) and E14.5 (f), E16.5 (g-i) forelimbs demonstrates LoxL3 marks muscle tips at multiple stages and in distinct regions. Immunostaining for LoxL3 (green) and Laminin (red) on E16.5 sections demonstrates LoxL3 is expressed inside the myofibers (j, arrows). In *LoxL3* mutant embryos, no LoxL3 staining is observed demonstrating antibody specificity (k-k''). FISH staining for *PDGFRα* (l, l'' green) and *MyoD1* (l', l'' red) along with immunostaining for MHC (l'' grey) on primary cultured cells following 48 hrs of differentiation demonstrates the two markers highlight distinct cells. Images shown are representative of at least three independent embryos or cell isolation experiments. Scale bars = 50 µm apart from b,c = 100 µm.

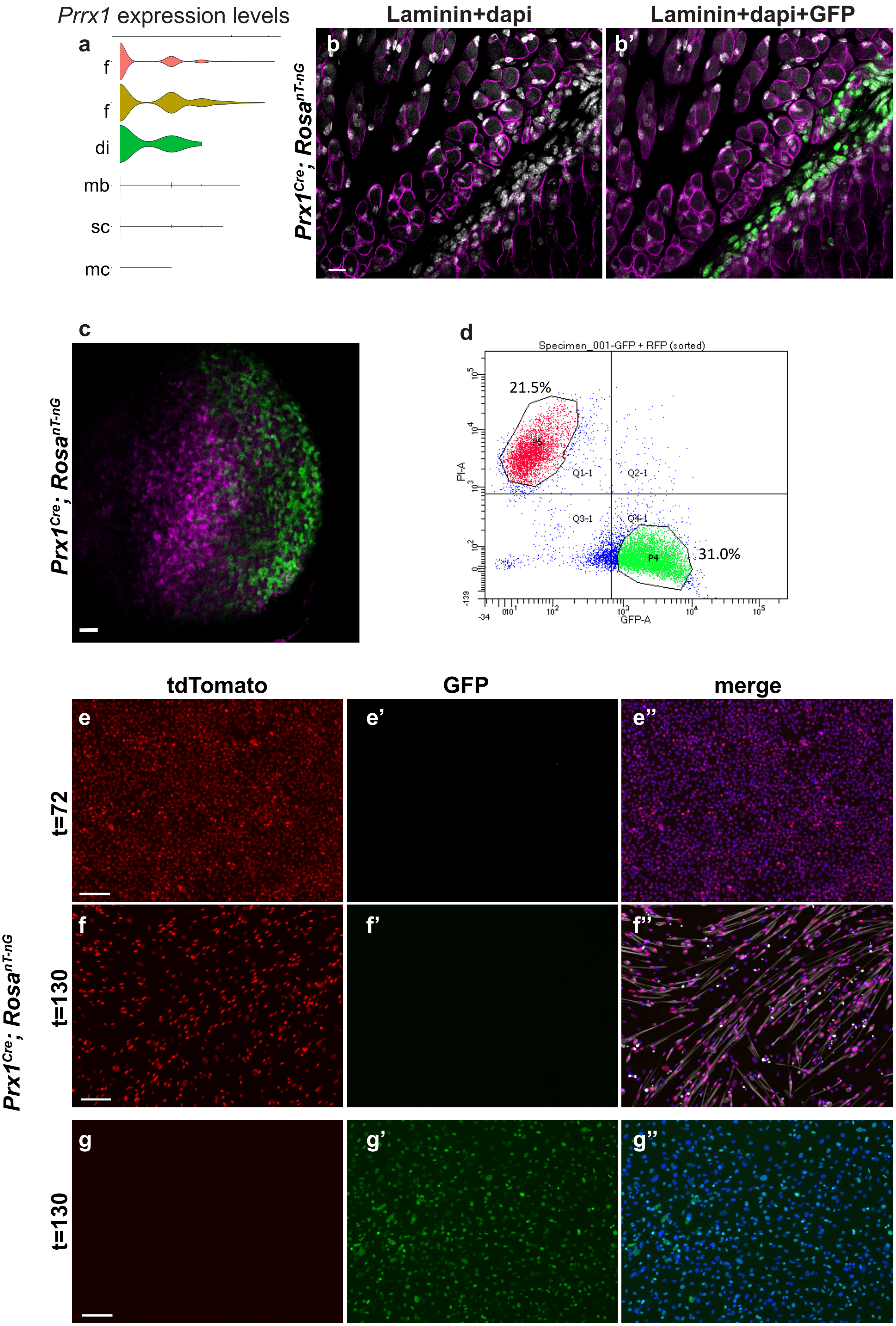

Supp. Figure 3

Supplementary Figure 3. ***Prx1^{Cre} is expressed in LPM-derived cells.*** Violin plot for *Prrx1* expression demonstrates it is expressed in LPM-derived cells, including dual identity cells, but not in myogenic cells (a). Immunostaining on transverse section of E18.5 *Prx1^{Cre}; Rosa^{nT-nG}* limb muscle demonstrating EGFP expressing nuclei are found in muscle facia but not in myofibers highlighted by sheathing Laminin (purple) (b-b'). Wholemount immunostaining for Pax3 (red) and EGFP (green) on E10.0 *Prx1^{Cre}; Rosa^{nT-nG}* limb bud showing myogenic progenitors (Pax3+ve) are EGFP negative (c). FACS plot demonstrates isolation of tdTomato or GFP positive cells (d) derived from *Prx1^{Cre}; Rosa^{nT-nG}* limb muscles. tdTomato (e,f) or EGFP (g) positive cells grown in culture for 72 or 130 hrs and stained MHC (grey) demonstrate Cre is not switched on following their isolation. Immunostaining images (b, b', e-g'') are representative results derived from at least three independent experiments. Scale bars = 10 µm apart from c= 100 µm.

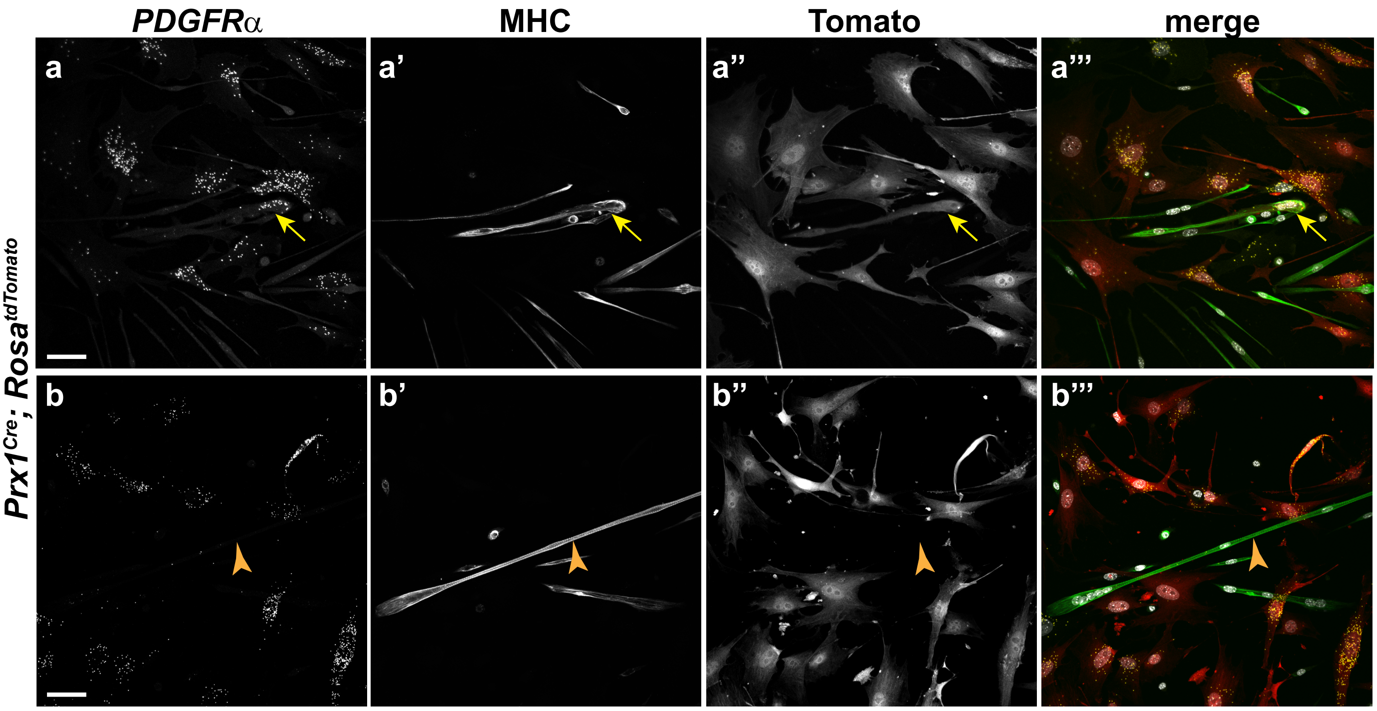

Supplementary Figure 4. ***LPM-derived cells contribute transcriptome to myofibers.*** Representative images (n=3 independent cell preparations from different litters) of FISH for *PDGFRα* on primary culture of myoblasts and interstitial cells derived from *Prx1^{Cre}; Rosa^{tdTomato}* following 48 hours of differentiation demonstrates that only in tdTomato-positive myofiber (a-a''') but not in tdTomato-negative (b-b''') fibers *PDGFRα* RNA is observed. Scale bars = 50 µm.

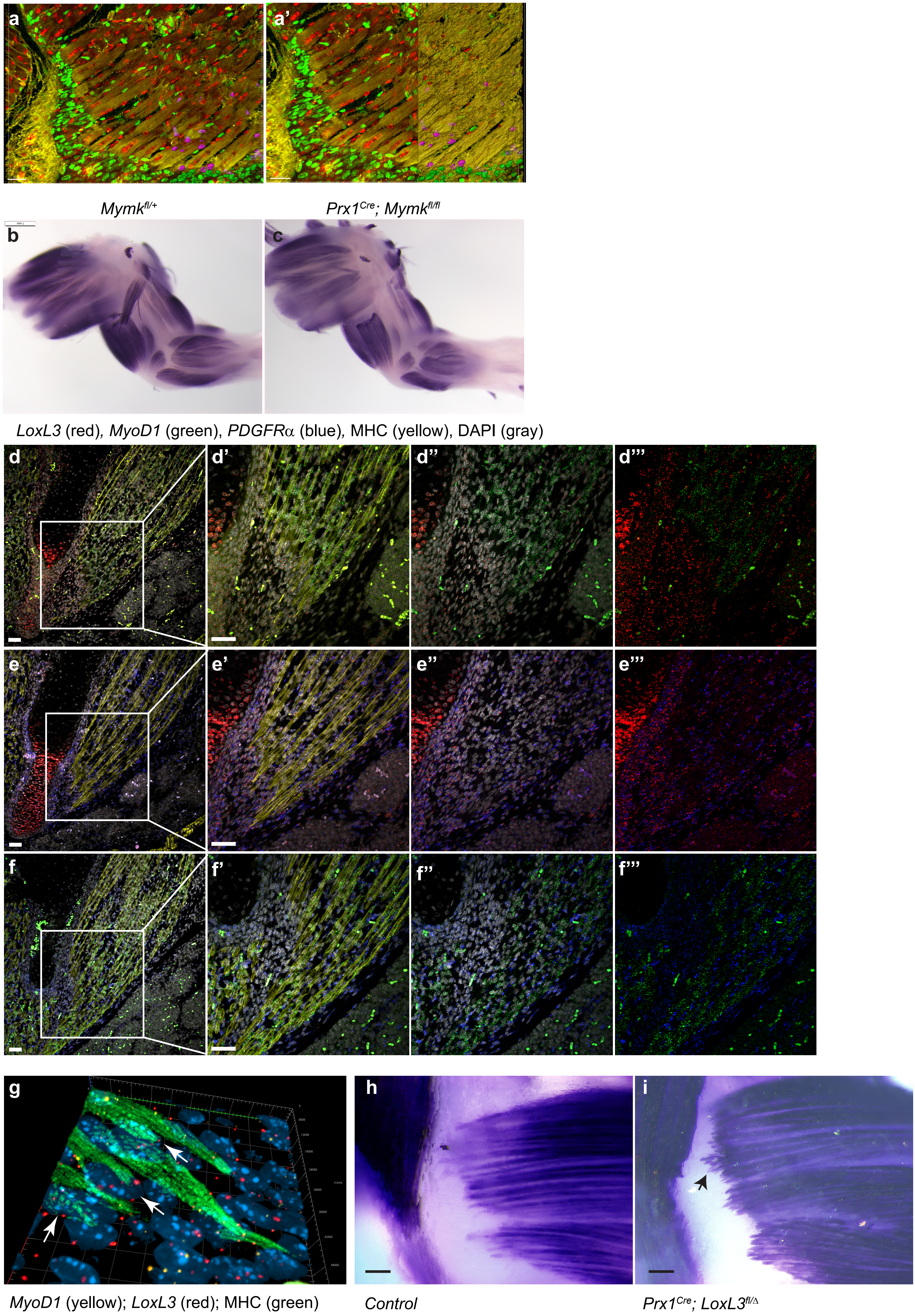

Supp. Figure 5

Supplementary Figure 5. ***Nuclei at myofiber tips express interstitial markers.*** Immunostaining for MHC (yellow), GFP and Tomato on a P0 *Prx1^{Cre}; Rosa26$^{nt\text{-}ng}$* limb (a) demonstrating process of IMARIS analysis to identify LPM-derived GFP positive cells inside myofibers (a' purple). MHC wholemount immunostaining of E16.5 *myomaker$^{fl/+}$* (b) or *Prx1^{Cre}; myomaker$^{fl/fl}$* (c) limbs. MHC immunostaining (yellow) and FISH for *LoxL3* (red) and *MyoD1* (green) (d), *LoxL3* (red) and *PDGFRα* (blue) (e), *MyoD1* (green) and *PDGFRα* (blue) (f). Tagged images show higher magnification of boxed regions in non-tagged ones (d-f'''). High magnification of myofibers tips (MHC, green) along with FISH for *LoxL3* (red) and *MyoD1* (yellow) (g). White arrows denote examples of *LoxL3* expressing nuclei that we are not able to determine if located in or out of myofiber. MHC immunostaining on (E16.5) control (h) and *Prx1^{Cre};LoxL3$^{fl/\Delta}$* (i) demonstrates MTJ defects are observed following *LoxL3* deletion in LPM (black arrow). IMARIS analyses (a,a'), immunostaining and FISH results (b-i) are representative results derived from at least three independent embryos from distinct litters. Unless stated, scale bars in a, a' = 30 µm, in d-f 50 µm and in h, i 100 µm.

PRIMER	SEQUENCE
LoxL3-5armWTF	GCCAGGGTGAAGTGAAAGAC
LoxL3-CritWTR	GATCTGGGATGCTGAAGACC
Tm1a-5mut-R1	GAACTTCGGAATAGGAACTTCG
CreORF REV	ATCCAGGTTACGGATATAGT
CreORF FWD	ATCCGAAAAGAAAACGTTGA
nT-nG_1	CCAGGCGGGCCATTTACCGTAAG
nT-nG_2	GGAGCGGGAGAAATGGATATG
nTnG_3	AAAGTCGCTCTGAGTTGTTAT
RosaTomato WT_FWD	AAGGGAGCTGCAGTGGAGTA
RosaTomato WT_REV	CCGAAAATCTGTGGGAAGTC

Supplementary Table 1. Primers used for genotyping the mice described throughout the work.
